# Genetic labeling reveals spatial and cellular expression pattern of neuregulin 1 in mouse brain

**DOI:** 10.1186/s13578-023-01032-4

**Published:** 2023-05-05

**Authors:** Chen-Yun Ding, Yan-Ting Ding, Haifeng Ji, Yao-Yi Wang, Xinwen Zhang, Dong-Min Yin

**Affiliations:** 1 Key Laboratory of Brain Functional Genomics, Ministry of Education and Shanghai, School of Life Science, East China Normal University, Shanghai, 200062 China; 2 Center of Implant Dentistry, School and Hospital of Stomatology, Liaoning Provincial Key Laboratory of Oral Diseases, China Medical University, Shenyang, 110002 China; 3 Shanghai Changning Mental Health Center, Affiliated to East China Normal University, Shanghai, 200335 China; 4 Laboratory Animal Centre, China Medical University, Shenyang, 110001 China; 5 NYU-ECNU Institute of Brain and Cognitive Science at NYU Shanghai, Shanghai, 200062 China

**Keywords:** Neuregulin 1, Cre-reporting mice, Olfactory bulb, Striatum, Cerebral cortex, Hippocampus, Hypothalamus, cerebellum, axon projection

## Abstract

**Background:**

Where the gene is expressed determines the function of the gene. Neuregulin 1 (Nrg1) encodes a tropic factor and is genetically linked with several neuropsychiatry diseases such as schizophrenia, bipolar disorder and depression. Nrg1 has broad functions ranging from regulating neurodevelopment to neurotransmission in the nervous system. However, the expression pattern of Nrg1 at the cellular and circuit levels in rodent brain is not full addressed.

**Methods:**

Here we used CRISPR/Cas9 techniques to generate a knockin mouse line (Nrg1^Cre/+^) that expresses a P2A-Cre cassette right before the stop codon of Nrg1 gene. Since Cre recombinase and Nrg1 are expressed in the same types of cells in Nrg1^Cre/+^ mice, the Nrg1 expression pattern can be revealed through the Cre-reporting mice or adeno-associated virus (AAV) that express fluorescent proteins in a Cre-dependent way. Using unbiased stereology and fluorescence imaging, the cellular expression pattern of Nrg1 and axon projections of Nrg1-positive neurons were investigated.

**Results:**

In the olfactory bulb (OB), Nrg1 is expressed in GABAergic interneurons including periglomerular (PG) and granule cells. In the cerebral cortex, Nrg1 is mainly expressed in the pyramidal neurons of superficial layers that mediate intercortical communications. In the striatum, Nrg1 is highly expressed in the Drd1-positive medium spiny neurons (MSNs) in the shell of nucleus accumbens (NAc) that project to substantia nigra pars reticulata (SNr). In the hippocampus, Nrg1 is mainly expressed in granule neurons in the dentate gyrus and pyramidal neurons in the subiculum. The Nrg1-expressing neurons in the subiculum project to retrosplenial granular cortex (RSG) and mammillary nucleus (MM). Nrg1 is highly expressed in the median eminence (ME) of hypothalamus and Purkinje cells in the cerebellum.

**Conclusions:**

Nrg1 is broadly expressed in mouse brain, mainly in neurons, but has unique expression patterns in different brain regions.

**Supplementary Information:**

The online version contains supplementary material available at https://doi.org/10.1186/s13578-023-01032-4.

## Background

Neuregulin 1 (Nrg1) encodes a tropic factor and is genetically associated with several neuropsychiatric diseases such as schizophrenia, bipolar disorder and depression [1–4]. NRG1 also shows neuroprotective effects in animal models of some neurological disorders such as ischemic stroke, epilepsy and Alzheimer’s disease [5–8]. NRG1 is a transmembrane protein and includes the N-terminal extracellular domain (ECD) and the C-terminal intracellular domain (ICD) [9]. The ECD of NRG1 binds with ErbB3 and ErbB4 receptors and activates the downstream signaling pathway. The NRG1-ErbB signaling is important for neurodevelopment such as myelination and GABAergic circuit formation [10–13]. NRG1-ErbB signaling also regulates synaptic transmission and plasticity such as GABA release and long-term potentiation (LTP) [14–18]. The ICD of NRG1 interacts with LIM kinase 1 (LIMK1) and sodium channel Nav1.1 to regulate glutamatergic transmission and neuronal excitability, respectively [19–22]. In addition, the ICD of NRG1 can bind with the gene promoter to regulate gene transcription [23–25].

Nrg1 mRNA has different splicing isoforms, each with a distinct N-terminal region. At least 6 types of splicing isoforms of Nrg1 mRNA were expressed throughout the brain [26]. Nrg1 is highly expressed in neurons in the mammalian brain [14, 27–29] although some studies reported Nrg1 was also expressed in glia cells [26, 27]. In the cerebral cortex and hippocampus, Nrg1 is mainly expressed in excitatory pyramidal neurons [6, 10, 27] while the ErbB4 receptor is specifically expressed by GABAergic interneurons [12, 30, 31].

The current knowledge about the function of NRG1 is mainly from the experiments with purified NRG1-ECD or ICD. The functions of endogenous Nrg1 and the Nrg1-expressing neurons are relatively unexplored [32, 33]. A prerequisite for addressing these issues is to understand the cellular expression pattern of Nrg1 and the axon projections of Nrg1-expressing neurons, i.e. expression pattern of Nrg1 at the cellular and circuit levels. The previous studies on the cellular expression pattern of Nrg1 include those from in situ hybridization (ISH), immunohistochemistry and translating ribosome affinity purification (TRAP) [14, 27, 28, 34]. However, the results from previous studies were sometimes controversial and did not reveal the expression pattern of Nrg1 at the circuit level.

The recently developed techniques of genetic labeling have enabled clear visualization of gene expression at the cellular level. These techniques usually include the use of a knockin mouse line which expresses the Cre recombinase immediately before the stop codon of the gene of interest, and a reporting mouse line which express fluorescent proteins in a Cre-dependent manner [31, 35–37]. Here we used CRISPR/Cas9 techniques to generate Nrg1^Cre/+^ knockin mice which express the Cre recombinase immediately before the stop codon of Nrg1 gene. By crossing the Nrg1^Cre/+^ mice with the Cre-dependent reporting mice (Ai14) [35], we generated the Nrg1-reporting mice which express tdTomato in Nrg1-positive cells. We then analyzed the cellular expression pattern of Nrg1 in the brain of Nrg1-reporting mice. We further injected Cre-dependent reporting AAV into the brain regions of adult Nrg1^Cre/+^ mice. Through this approach, we explored Nrg1 expression pattern and the axon projections of Nrg1-positive neurons in adult mouse brain. These results provide a fundamental framework for the study of Nrg1 function in the rodent brain. Moreover, the Nrg1^Cre/+^ mice generated here may represent a useful tool to study the function of neuronal populations expressing Nrg1.

## Methods

### Generation of Nrg1^Cre/+^ knockin mice

**Fig. 1:**
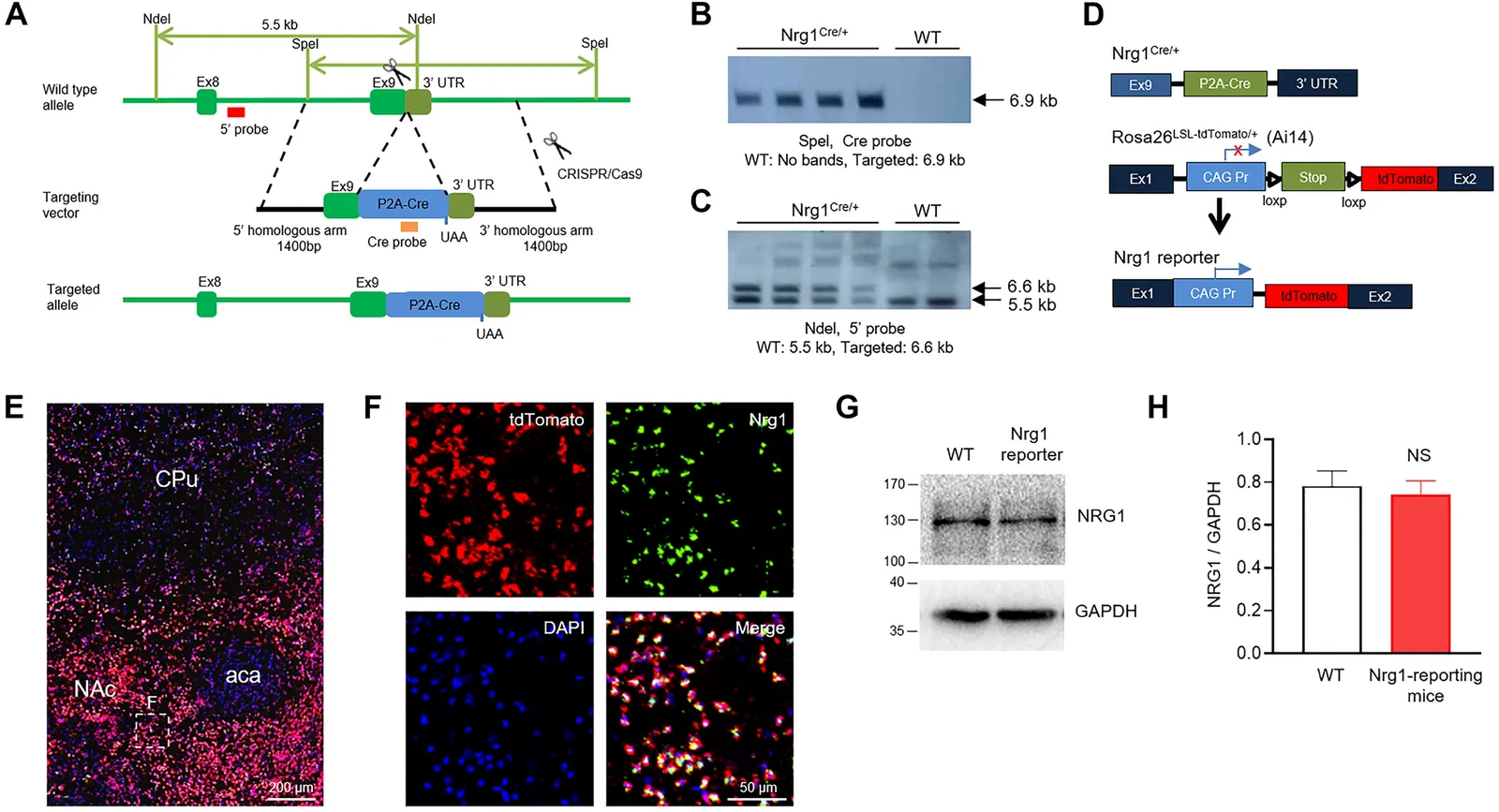
Generation and validation of Nrg1-reporting mice. **A** Schematic diagram of the gene targeting strategy to insert the P2A-Cre cassette immediately before the stop codon of the Nrg1 locus, between exon 9 and 3’ untranslational region (3’UTR). The p2A peptide will be cleaved and two independent protein NRG1 and CRE will be expressed. **B** Southern blot screen for Nrg1^Cre/+^ mice using Spel-digested genomic DNA and the Cre probe indicated in panel A. The targeted Nrg1 allele will yield a DNA fragment of 6.9 kb. **C** Southern blot screen for Nrg1^Cre/+^ mice using Ndel-digested genomic DNA and the 5’ probe indicated in panel A. The wild-type and targeted Nrg1 allele will yield a DNA fragment of 5.5 kb and 6.6 kb, respectively. **D** The breeding strategy to get the Nrg1-reporting mice. The female Nrg1^Cre/+^ mice were crossed with the male Rosa26^LSL−tdTomato/+^ (Ai14) mice to get the Nrg1^Cre/+^; Rosa26^LSL−tdTomato/+^ mice, i.e., the Nrg1-reporting mice. **E** Double fluorescence in situ hybridization (dFISH) of tdTomato and Nrg1 mRNA in the striatum of Nrg1-reporting mice. The arrows indicate Nrg1-positive granule cells in the OB. Scale bar, 200 μm. **F** The enlarged image from the rectangle in panel E. Scale bar, 50 μm. **G–H** Similar NRG1 protein levels in the striatum between WT and Nrg1-reporting mice. G, representative western blots, the total lysates of striatum from WT and Nrg1-reporting mice were probed with anti-NRG1 and anti-GAPDH antibodies. H, quantification results. NS, not significant, n = 3, unpaired t-test

A P2A-Cre cassette was placed between the coding sequence of axon 9 (immediately before the stop codon) and the 3’ UTR of mouse Nrg1 gene (Fig. 1A). The P2A is a small peptide originally from porcine teschovirus and can be self-cleaved at its C terminus in all eukaryotic cells [38]. In the Nrg1^Cre/+^ knockin mice, a fusion protein NRG1-P2A-Cre are transiently expressed but NRG1 and Cre proteins are ultimately separated after P2A-mediated cleavage. The detail of using CRISPR/Cas9 techniques to do gene editing in mice was described previously [39]. Briefly, sgRNA, Cas9 mRNA, and targeting vectors were injected into the cytoplasm of one-cell stage embryos through the injection needle. Injections were performed using an Eppendorf transferMan NK2 micromanipulator. Injected zygotes were transferred into pseudopregnant female C57BL/6 mice after 2-h culture in KSOM medium. This strain was generated in Beijing Biocytogen Co., Ltd., and maintained on a C57BL/6 background. The F0 chimera mice were crossed with wild type (WT) mice to get the germline transmission F1 mice. The correct targeting of the Nrg1^Cre/+^ in F1 mice was confirmed by southern blot and gene sequencing. The primers for genotyping the WT-Nrg1 and Nrg1::Cre allele are as follows: forward: 5’atggccacattgccaataggttgga3’; reverse: 5’tgctctcgacataacataacataaaggca3’. The PCR products for WT-Nrg1 and Nrg1::Cre alleles were 514 and 1633 bp, respectively.

### Generation of Nrg1-reporting mice

The F1 Nrg1^Cre/+^ mice were backcrossed with WT mice to get the F2 Nrg1Cre/ + mice. The F2 Nrg1^Cre/+^ mice were crossed with heterozygous Ai14 mice (Rosa26^LSL−tdTomato/+^ mice, JAX stock #007908) [35] to get the Nrg1^Cre/+^;Rosa26^LSL−tdTomato/+^ mice (abbreviated to Nrg1-reporting mice). The tdTomato was specifically expressed in the cells with Cre activity which was controlled by the Nrg1 promoter. Cre dependent expression of tdTomato has frequently been used to study the expression pattern of genes with a high temporal and spatial resolution [31, 35–37]. Mice were housed at 23 °C with a 12 h light/dark cycle and food and water available ad libitum. Both female and male Nrg1-reporting mice were used and showed similar Nrg1 expression pattern in forebrain regions. For the quantification, at least three different mice were used for each group. The Nrg1-reporting mice were crossed with Gad67-GFP mice [40] to visualize Nrg1 expression in GABAergic interneurons. All experimental procedures were reviewed and approved by the institutional animal care and use committee of East China Normal University.

### Fluorescence in situ hybridization (FISH)

FISH for mRNA expression was performed manually using the RNAscope Multiplex Fluorescent Reagent Kit v2 (Advanced Cell Diagnostics, Inc., Hayward, CA, USA) following the manufacturer’s instruction. The RNAscope probes targeting Nrg1, tdTomato and Drd1 were from Advanced Cell Diagnostics. The reference numbers are as follows: 418181-C3 for Nrg1 probe, 317041 for tdTomato probe, 406491 for Drd1 probe.

### Immunofluorescence

After being anesthetized with euthatal (from Merck) (60 mg/kg), the Nrg1-reporting mice (2 months old) were transcardially perfused with PBS (2 ml/g of body weight), followed by 4% PFA in PBS. Brains were harvested, incubated in 4% PFA overnight, and dehydrated at 4 °C in two steps with 20% and 30% sucrose in PBS. Brains were frozen in OCT (catalog #14-373-65; Fisher) and sectioned into 40 µm slices on a cryostat microtome (Bosch Microm HM550) at − 20 °C. Brain slices were permeabilized with 0.3% TritonX 100 and 5% BSA in PBS and incubated with primary antibodies at 4 °C overnight. The brain slices were not treated with Triton- × 100 when staining with anti-GAD67 antibodies. After washing with PBS for three times, samples were incubated with Alexa Fluor-488 or -647 secondary antibodies (1:1000, ThermoFisher Scientific) for 1 h at room temperature. Samples were mounted with Vectashield mounting medium (Vector) and images were taken by Leica TCS SP8 confocal microscope. The following primary antibodies were used: rabbit anti-NeuN (1:500, Abcam, ab177487), rabbit anti-TH (1:250, Millipore, MAB152), mouse anti-NRGN (1:200, R&D, MAB7947), mouse anti-PV (1:500, Sigma, P3088), mouse anti-GFAP (1:250, Millipore, MAB360); rabbit anti-S100β (1:200, Abcam, Ab52642) and mouse anti-GABA (1:1000, Invitrogen, PA5-32241).

### Analysis of Nrg1-positive cells in brain slices

Images were taken on a Leica TCS SP8 scanning confocal microscopy. Unbiased stereology (Tissue Gnostics, Vienna, Austria) was applied to quantification of Nrg1^+^ cell number in brain slices [36, 37]. The detailed methods for cell number counting is available on the website (https://tissuegnostics.com/products/single-cell-analysis/tissuequest). To compare the Nrg1 expression among different brain regions of adult mice, we count the density of Nrg1^+^ cells. The density was calculated from eight continuous sections in Z-stack. Coronal brain slices from 4.3 to 3.9 mm relative to bregma were used to study OB, 1.7 to 1.4 mm relative to bregma were used to access striatum, − 1.5–− 1.8 mm relative to bregma were used to study primary somatosensory cortex (SS1), − 1.7–− 1.9 mm relative to bregma were used to investigate dorsal hippocampus.

### Western blot

Homogenates of striatum from WT and Nrg1-reporting mice were prepared in RIPA buffer containing 50 mM Tris–HCl, pH 7.4, 150 mM NaCl, 2 mM EDTA, 1% sodium deoxycholate, 1% SDS, 1 mM PMSF, 50 mM sodium fluoride, 1 mM sodium vanadate, 1 mM DTT, and protease inhibitors cocktails. Homogenates were resolved on SDS/PAGE and transferred to nitrocellulose membranes, which were incubated in the TBS buffer containing 0.1% Tween-20 and 5% milk for 1 h at room temperature before the addition of primary antibody for incubation overnight at 4 °C. After wash, the membranes were incubated with HRP-conjugated secondary antibody in the same TBS buffer for 1 h at room temperature. Immunoreactive bands were visualized by ChemiDocTM XRS + Imaging System (BIO-RAD) using enhanced chemiluminescence (Pierce) and analyzed with Image J (NIH). The following antibodies were used: rabbit anti-NRG1 (1:1000, Cell Signaling Technology, #2573) and mouse anti-GAPDH (1:8000, Arigo, ARG10112).

### Stereotaxic injection of AAV into brain regions

The Cre-reporting AAV (pAAV-EF1α-loxp-stop-loxp-tdTomato-WPRE-polyA, titer 1 × 10^13^/µl) was generated by Obio Technology (Shanghai) Corp., Ltd. Adult Nrg1^Cre/+^mice (2-month-old) were anesthetized with euthatal (60 mg/kg, i.p. injection) and headfixed in a stereotaxic device (RWD life science). Each injection used 0.5 μl AAV and took 10 min. After injection, the glass pipette was left in place for 10 min to facilitate diffusion of the virus. The injection sites were examined at the end of the experiments, and animals with incorrect injection site were excluded from the data analysis. Injection coordinates are as follows: anteroposterior (AP) 3.92 mm, dorsoventral (DV) 1.75 mm, mediolateral (ML) 0.75 mm relative to bregma for olfactory bulb; AP − 1.82 mm, DV 1.90 mm, and ML 1.12 mm relative to bregma for dentate gyrus; AP + 1.42 mm, DV 5.00 mm, and ML 1.15 mm relative to bregma for striatum; AP − 6.24 mm, DV 2.00 mm, and ML 0 mm relative to bregma for cerebellum. Four weeks after AAV injection, mice were subjected to experiments. All surgery was conducted with aseptic technique.

### Statistics

All the data were shown as mean ± SEM. Comparisons between two groups were made using unpaired t test. Comparisons between three or more groups were made using one-way ANOVA analysis followed by Tukey’s post hoc test. Analysis of the data from different layers of multiple cortical regions were performed using two-way-ANOVA followed by Tukey’s multiple comparison test. The p values were provided in the figure legends and were significant when smaller than 0.05. The statistical analysis was performed with the software of GraphPad Prism 8.

## Results

### Generation and validation of Nrg1-reporting mice

We first generated Nrg1^Cre/+^ knockin mice where a P2A-Cre cassette was placed right before the stop codon (UAA) of Nrg1 gene (Fig. 1A) (see details in the Method). In the Nrg1^Cre/+^ knockin mice, the expression of Cre is under the control of endogenous Nrg1 promoter and thus Cre and Nrg1 are expressed in the same types of cells. The results from Southern blot validated the insertion of P2A-Cre into the target locus, indicating that the generation of Nrg1^Cre/+^ mouse line is methodologically reliable (Fig. 1B, C). To acquire the Nrg1-reporting mice, the Nrg1^Cre/+^ mice were crossed with the Cre-reporting Ai14 mice [35] (Fig. 1D). The Nrg1-reporting mice develop normally and are fertile. To verify that tdTomato is specifically and faithfully expressed in Nrg1-positive cells from Nrg1-reporting mice, we performed FISH to detect the Nrg1 mRNA in the nucleus accumbens (NAc) where the expression of Nrg1 is high [41]. As shown in Fig. 1E, F, Nrg1 mRNA was well colocalized with tdTomato in the NAc of Nrg1-reporting mice. Most tdTomato-positive cells (94 ± 1.2%) in the NAc of Nrg1-reporting mice express Nrg1 mRNA. In addition, the protein levels of NRG1 in the striatum are similar between the Nrg1-reporting mice and their wild type (WT) littermates (Fig. 1G, H). Thus, we validated on mRNA levels that tdTomato from Nrg1-reporting mice can be used as a faithful indicator of Nrg1-positive cells.

### Expression pattern of Nrg1 in the olfactory bulb

**Fig. 2:**
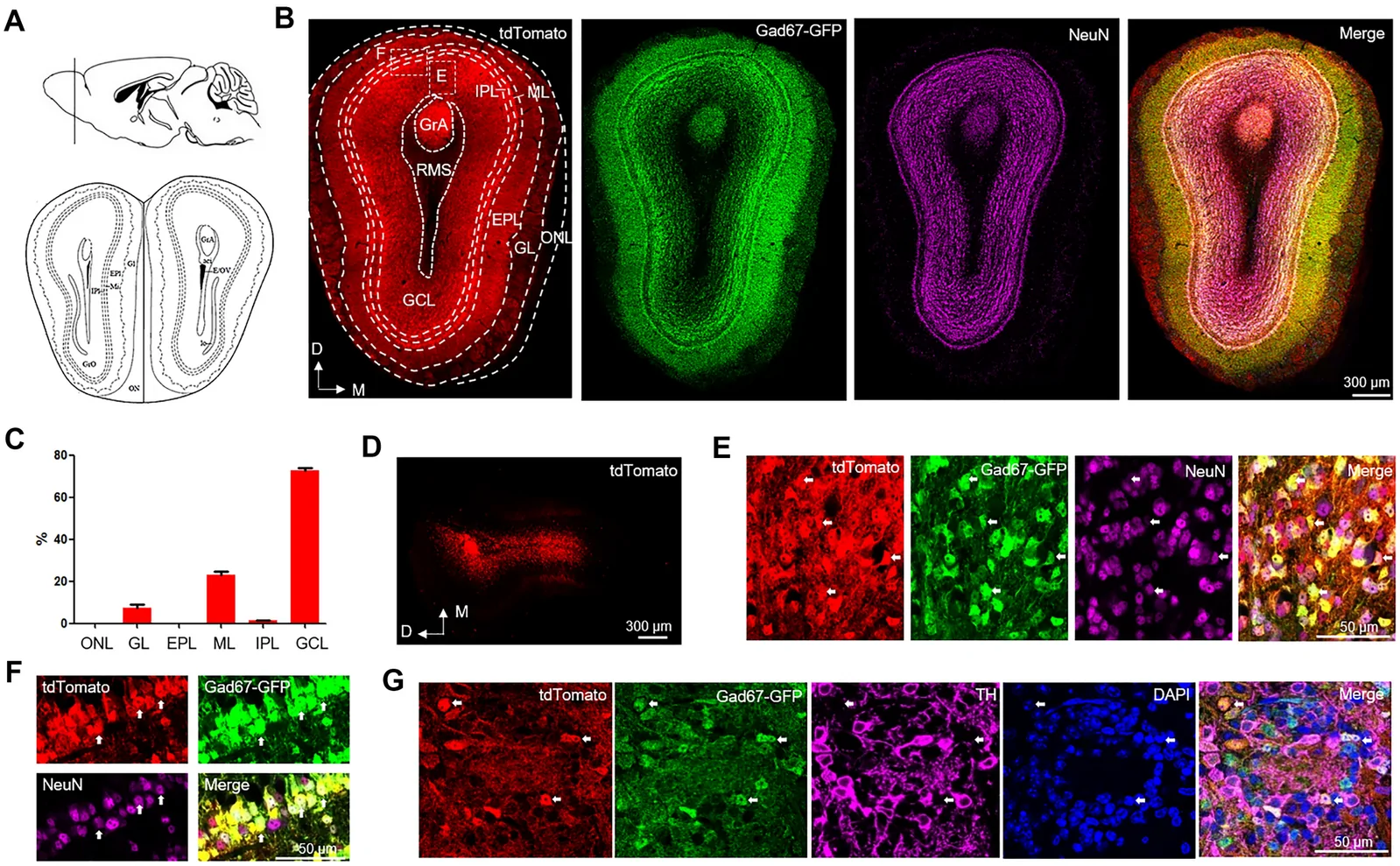
Expression pattern of Nrg1 in the olfactory bulb. **A** Diagram of mouse brain sagittal section (top) and coronal section (bottom). The line in the sagittal section diagram indicates the position of the coronal section. **B** Fluorescent images of tdTomato, Gad67-GFP and NeuN in the OB of 2-month-old Nrg1-reporting mice. D, dorsal. M, medial. Scale bar, 300 μm. **C** The percentage of Nrg1-positive cells in different layers among total Nrg1-positive cells in the OB. P < 0.0001, n = 3 mice (9 slices), one-way-ANOVA. **D** Expression of tdTomato after injection of Cre-reporting AAV into the GCL of 2-month-old Nrg1^Cre/+^ mice. Four weeks after AAV injection, the brain slices were collected and analyzed. Scale bar, 300 μm. **E** Fluorescent images of tdTomato, Gad67-GFP and NeuN in the GCL, from the rectangle E in panel B. Arrows indicate Nrg1-positive granular cells in the GCL. Scale bar, 50 μm. **F** Fluorescent images of tdTomato, Gad67-GFP and NeuN in the ML, from the rectangle F in panel B. Arrows indicate Nrg1-positive GABAergic interneurons in the ML. Scale bar, 50 μm. **G** Fluorescent images of tdTomato, Gad67-GFP, TH and DAPI in GL. Arrows indicate Nrg1-positive PG cells expressing Gad67-GFP but not TH. Scale bar, 50 μm. The Nrg1-reporting mice were crossed with Gad67-GFP mice to visualize the expression of Nrg1 in GABAergic interneurons. ONL: olfactory nerve layer; GL: glomerular layer; EPL: external plexiform layer; ML: mitral cell layer; IPL: internal plexiform layer; GCL: granular cell layer; GrA: granule cell layer of the accessory olfactory bulb; RMS; rostral migratory stream

We analyzed the densities of Nrg1-positive cells in several brain regions of Nrg1-reporting mice, and revealed that the olfactory bulb (OB) had the most abundant Nrg1-positive cells (Additional file 1: Fig. S1). The OB is the first brain region where olfactory information is processed. The OB can be divided into olfactory nerve layer (ONL), glomerular layer (GL), external plexiform layer (EPL), mitral layer (ML), internal plexiform layer (IPL) and granular cell layer (GCL) (Fig. 2A, B). The Nrg1-positive cells were most abundant in the GCL, accounting for 73.4 ± 2.3% of the total Nrg1-positive cells, followed by the ML and GL, accounting for 23.6 ± 3.0% and 7.27 ± 2.8% of the total Nrg1-positive cells, respectively (Fig. 2B, C). Nrg1 is also highly expressed in the granule cell layer of the accessory olfactory bulb (GrA) (Fig. 2B). However, few Nrg1-positive cells are distributed in ONL, EPL, IPL and rostral migratory stream (RMS) (Fig. 2B, C). Adult expression of Nrg1 in the GCL of OB has not been well reported, which raises the possibility that the tdTomato signal we observed may arise from the transient expression of Nrg1 in the early development instead of in the adulthood. To address this possibility, we injected Cre-reporting AAV into the GCL of 2-month-old WT and Nrg1^Cre/+^ mice. After injecting AAV into the GCL of WT mice, tdTomato did not appear in granule cells (data not shown). However, when AAV was injected into the GCL of Nrg1^Cre/+^ mouse, the expression of tdTomato was observed in granule cells (Fig. 2D). These results indicated that Nrg1 is, indeed, expressed in the granule cells of OB in adult mice.

We crossed the Nrg1-reporting mice with Gad67-GFP mice to investigate whether Nrg1 is expressed in the GABAergic interneurons. In the GCL and ML, Nrg1 is expressed in the GABAergic interneurons as indicated by the co-localization of tdTomato and GFP (Fig. 2E, F). In the GL, Nrg1 is expressed in the periglomerular (PG) cells that are GABAergic interneurons as evidenced by the co-localization of tdTomato with GFP (Fig. 2G). In contrast, tdTomato was not co-localized with tyrosine hydroxylase (TH, a protein marker for short-axon cells in the GL), which suggests that Nrg1 is not expressed in short-axon cells in the GL (Fig. 2G). Together, these results demonstrate that Nrg1 is mainly expressed in GABAergic interneurons in mouse OB.

### Expression pattern of Nrg1 in the cerebral cortex

**Fig. 3:**
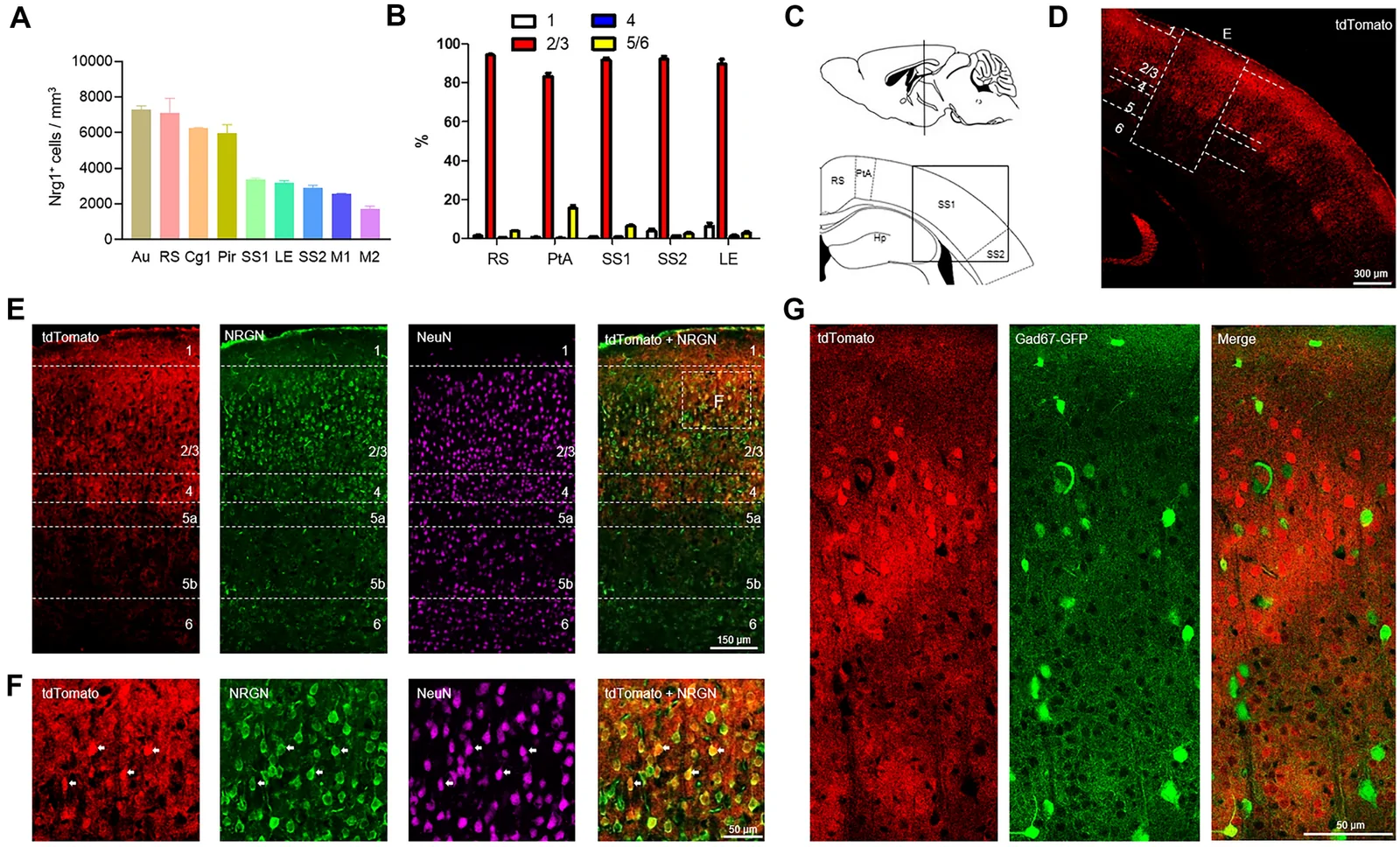
Expression pattern of Nrg1 in the cerebral cortex. **A** The densities of Nrg1-positive cells in different regions of cerebral cortex from 2-month-old Nrg1-reporting mice. P < 0.0001, n = 3 mice (6 slices) for Au and LE, n = 3 mice (9 slices) for other cortical regions, one-way-ANOVA. **B** The percentage of Nrg1-positive cells in different layers among total Nrg1-positive cells from multiple cortical regions. Layer factor P < 0.0001, region factor P > 0.99, n = 3 mice (9 slices) for each cortical region, two-way-ANOVA. **C** Diagram of mouse brain sagittal section (top) and coronal section (bottom). The line in the sagittal section diagram indicates the position of the coronal section. The rectangle indicates the brain region shown in panel D. **D** Fluorescent images of tdTomato in the SS1. Scale bar, 300 μm. **E** Fluorescent images of tdTomato, NRGN and NeuN, from the rectangle in panel D. Scale bar, 150 μm. **F** Fluorescent images of tdTomato, NRGN and NeuN, enlarged from the rectangle in panel E. Arrows indicate Nrg1-positive pyramidal neurons. Scale bar, 50 μm. **G** Fluorescent images of tdTomato and gad67-GFP in the SS1. Scale bar, 50 μm. Au: auditory cortex; RS: retrosplenial cortex; Cg1: cingulate cortex, area 1; Pir: piriform cortex; SS1: primary somatosensory cortex; LE: lateral entorhinal cortex; SS2: secondary somatosensory cortex; M1: primary motor cortex; M2: secondary motor cortex; PtA: parietal association cortex

The cerebral cortex is the outer most structure of the mammalian brain having a distinct six-layer composition. Here, high-level processing occurs for many processes including motor control, sensory perception, attention, and memory. Nrg1-positive cells were widely distributed in different cortical regions. The density of Nrg1-positive cells is relatively higher in the cingulate cortex, area 1 (Cg1), retrosplenial cortex (RS), piriform cortex (Pir) and auditory cortex (Au) than that in the primary motor cortex (M1), secondary motor cortex (M2), primary somatosensory cortex (SS1), secondary somatosensory cortex (SS2) and lateral entorhinal cortex (LE) (Fig. 3A). Nrg1 was expressed in different layers of cerebral cortex. However, the percentage of Nrg1-positive cells in the superficial layers is significantly higher than deep layers in multiple cortical regions (Fig. 3B). In the following study, we take the cortical region of SS1 as an example.

In the SS1, tdTomato signal was mainly distributed in the superficially layer 2–3 (Fig. 3D, E). The tdTomato signal was localized in some fiber-like structure and cell bodies that express NeuN (a pan neuronal marker) and neurogranin (NRGN, a pyramidal neuronal marker), but not all NRGN-positive cells expressed Nrg1 (3E and 3F). We further crossed the Nrg1-reporting mice with Gad67-GFP mice to investigate whether Nrg1 is expressed in the GABAergic interneurons in the SS1. As shown in Fig. 3G, we did not observe the co-expression of tdTomato and GFP in the same cells, indicating low expression of Nrg1 in GABAergic interneurons in the SS1. Overall these data suggest that Nrg1 is mainly expressed in part of pyramidal neurons in the superficial layers of SS1.

### Expression pattern of Nrg1 in the striatum

**Fig. 4:**
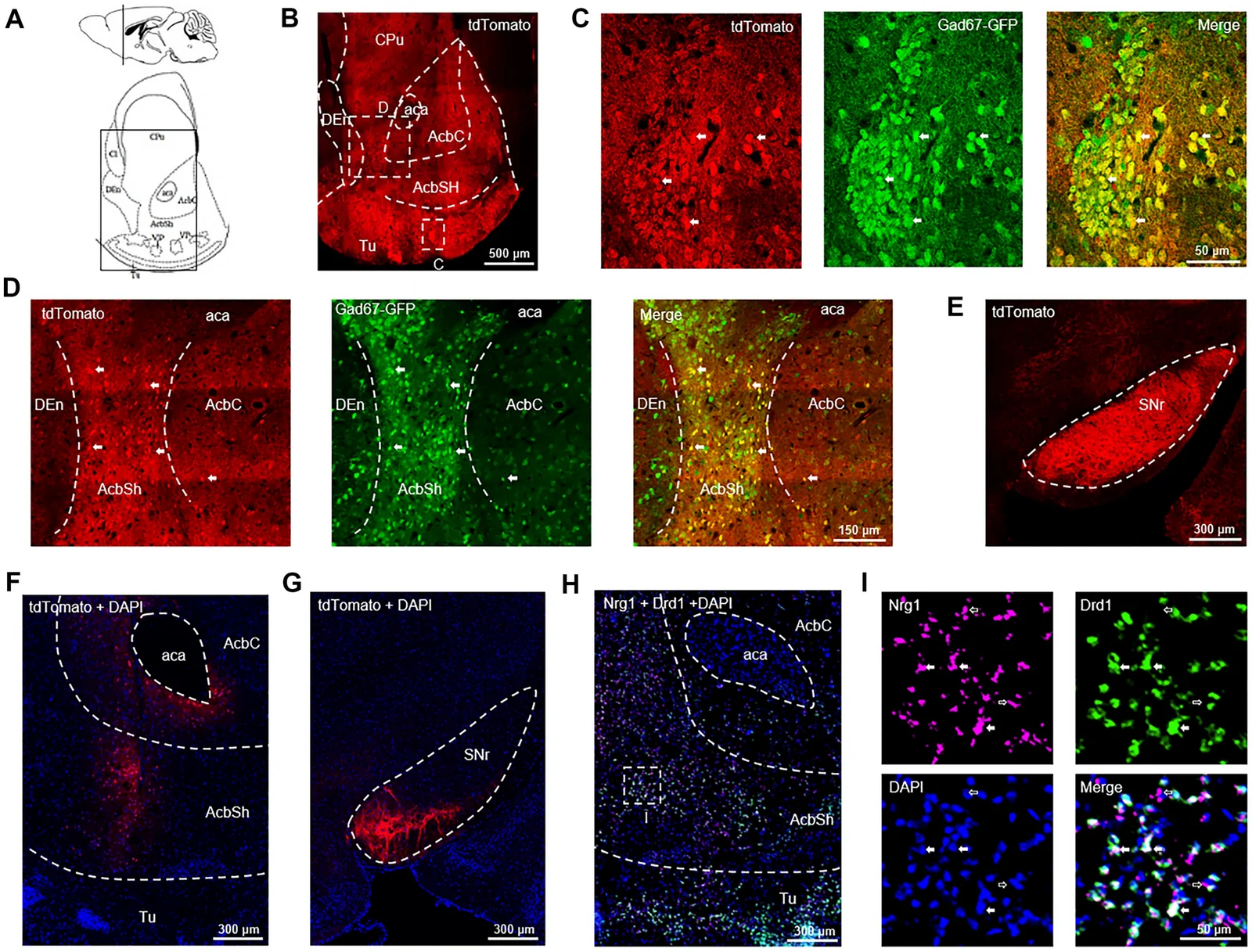
Expression pattern of Nrg1 in the striatum. **A** Diagram of mouse brain sagittal section (top) and coronal section (bottom). The line in the sagittal section diagram indicates the position of the coronal section. The rectangle indicates the brain region shown in panel B. **B** Expression of tdTomato in the striatum of 2-month-old Nrg1-reporting mice. Scale bar, 500 μm. **C–D** Fluorescent images of tdTomato and Gad67-GFP in the Tu **C** and AcbSH **D** from the rectangles in panel B. Arrows indicate Nrg1-positive cells which are GABAergic MSNs. Scale bar, 50 μm in panel C, 150 μm in panel D. The Nrg1-reporting mice were crossed with Gad67-GFP transgene mice to visualize the expression of Nrg1 in GABAergic MSNs. **E** Expression of tdTomato in axon fiber-like structures in the SNr. Scale bar, 300 μm. **F–G** Expression of tdTomato in the NAc **F** and SNr (**G** after injection of Cre-reporting AAV into the NAc of 2-month-old Nrg1^Cre/+^ mice. Four weeks after AAV injection, the brain slices were collected and analyzed. Scale bar, 300 μm. **H** Double fluorescence in situ hybridization (dFISH) of Nrg1 and Drd1 mRNA in the striatum of WT mice. Scale bar, 300 μm. **I** The enlarged image from the rectangle in panel H. The solid arrows indicate Nrg1-positive cells expressing Drd1. The empty arrows indicate Nrg1-positive cells not expressing Drd1. Scale bar, 50 μm. CPu: caudate putamen; Den: dorsal endopiriform nucleus; aca: anterior commissure, anterior part; AcbC: accumbens nucleus, core; AcbSH: accumbens nucleus, shell; Tu: olfactory tubercle; SNr: substantia nigra, reticular part

Apart from locomotion, the striatum has been closely implicated in regulating reward and motivation. The dorsal and ventral part of striatum are composed of the caudate putamen (CPu) and NAc, respectively. Most neurons in the striatum are GABAergic medium spiny neurons (MSNs) which receive dopaminergic input from substantia nigra (SN) and ventral tegmental area (VTA). In the striatum, Nrg1-positive cells were mainly distributed in the regions of ventral striatum including the shell of NAc (AcbSH) and olfactory tubercle (Tu) (Fig. 4A–D). By contrast, the Nrg1-positive cells are quite sparse in the core of NAc (AcbC) and CPu (Fig. 4B, D).

We crossed Nrg1-reporting mice with Gad67-GFP transgene mice to verify whether Nrg1 is expressed in the GABAergic MSNs in the striatum. All Nrg1-positive cells in the Tu and AcbSH are GABAergic MSNs but not vice versa because all the tdTomato-positive cells express GFP while only some of the GFP-positive cells express tdTomato (Fig. 4C, D). One type of MSNs express dopamine D1 receptor (Drd1) and directly project to substantia nigra pars reticulata (SNr), which is called the direct pathway [42]. The other type of MSNs express dopamine D2 receptor (Drd2) and connect with SNr indirectly through external globus pallidus (GPe) and subthalamic nucleus, which is referred to indirect pathway [42]. Intriguingly, we found strong expression of axon fiber-like tdTomato signal in the SNr (Fig. 4E), which suggest that Nrg1-positive cells in the NAc have axon projections to the SNr. To verify this hypothesis, we performed stereotaxic injection of Cre-reporting AAV into the NAc of 2-month-old Nrg1^Cre/+^ mice. Four weeks after AAV injection, we found tdTomato-positive cells in the NAc and tdTomato-positive axon fibers in the SNr (Fig. 4F, G). These results indicate that Nrg1-positive cells directly send their axons to SNr, i.e., Nrg1 is expressed in the MSNs of the direct pathway. We further performed FISH to investigate whether Nrg1 and Drd1 (a maker for the MSNs in the direct pathway) are expressed in the same type of cells. This is indeed the case because most Nrg1-positive cells also expressed Drd1 (Fig. 4H and 4I). Together, these results demonstrate that Nrg1 is mainly expressed in the MSNs of the direct pathway in the NAc.

### Expression pattern of Nrg1 in the hippocampus

**Fig. 5:**
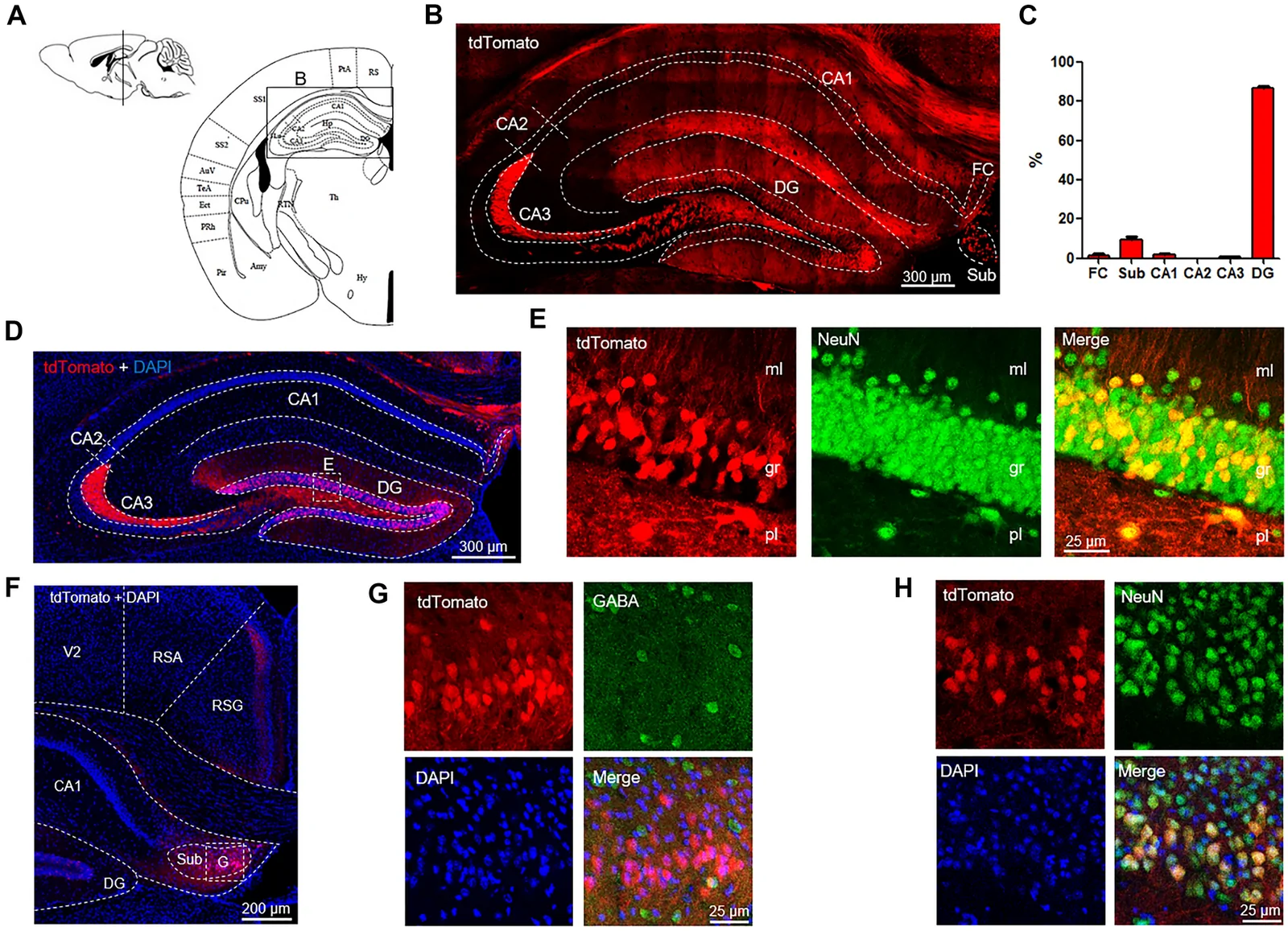
Expression pattern of Nrg1 in the hippocampus. **A** Diagram of mouse brain sagittal section (top) and coronal section (bottom). The line in the sagittal section diagram indicates the position of the coronal section. The rectangle indicates the brain region shown in panel B. **B** Expression of tdTomato in the dorsal hippocampus of 2-month-old Nrg1-reporting mice. Scale bar, 300 μm. **C** The percentage of Nrg1-positive cells in different subregions of dorsal hippocampus among total Nrg1-positive cells. P < 0.0001, n = 3 mice (9 slices) for FC, n = 6 mice (12 slices) for other regions, one-way-ANOVA. **D** Expression of tdTomato in DG and CA3 region after injection of Cre-reporting AAV into the DG of 2-month-old Nrg1^Cre/+^ mice. Four weeks after AAV injection, the brain slices were collected and analyzed. Scale bar, 300 μm. **E** Fluorescent images of tdTomato and NeuN from the rectangle in panel D. Scale bar, 25 μm. **F** Expression of tdTomato in Sub and RS region after injection of Cre-reporting AAV into the Sub of 2-month-old Nrg1^Cre/+^ mice. Four weeks after AAV injection, the brain slices were collected and analyzed. Scale bar, 200 μm. **G** Fluorescent images of tdTomato and GABA from the rectangle in panel F. Scale bar, 25 μm. **H** Fluorescent images of tdTomato, NeuN and DAPI in the Sub of hippocampus. Scale bar, 25 μm. FC: fasciola cinereum; Sub: subiculum; CA1: cornu ammonis 1; CA2: cornu ammonis 2; CA3: cornu ammonis 3; DG: dentate gyrus; ml: molecular layer; gr: granular layer; pl: polymorph layer; RSA: retrosplenial agranular cortex; RSG: retrosplenial granular cortex; V2: secondary visual cortex

The hippocampus, located beneath the cerebral cortex, is critically involved in learning and memory, in addition to spatial navigation. The hippocampus can be divided into cornu ammonis (CA) 1, 2, 3 areas, the dentate gyrus (DG), subiculum (Sub) and fasciola cinereum (FC) (Fig. 5A). The tdTomato-positive cells were mainly distributed in the DG and Sub but were very sparse in the CA and FC (Fig. 5B, C). In addition, we observed axon fiber-like tdTomato signals in the CA3 region which resemble the mossy fiber from DG granule cells (Fig. 5B). These results suggest that Nrg1 is mostly expressed in the DG and Sub of mouse hippocampus.

To solidify the expression of Nrg1 in the DG and Sub of adult mouse hippocampus, we performed stereotaxic injection of Cre-reporting AAV into the DG and Sub of 2-month-old Nrg1^Cre/+^ mice, respectively. Four weeks after AAV injection, we found tdTomato-positive cells in the DG and Sub (Fig. 5D, F), which verify the expression of Nrg1 in the DG and Sub of adult mouse hippocampus. Nrg1 is expressed by the mature granule neurons in the DG evidenced by the co-localization of tdTomato and NeuN, a protein marker for mature neurons (Fig. 5E). We did not observe the colocalization of tdTomato with GFAP or S100β (two protein markers for astrocytes) in the DG (Additional file 2: Fig. S2), indicating that Nrg1 is rarely expressed in astrocytes. In the Sub, Nrg1 is probably expressed in the excitatory pyramidal neurons since tdTomato was co-localized with NeuN (a pan neuronal marker) but not GABA (a GABAergic neuronal marker) (Fig. 5G, H).

**Fig. 6:**
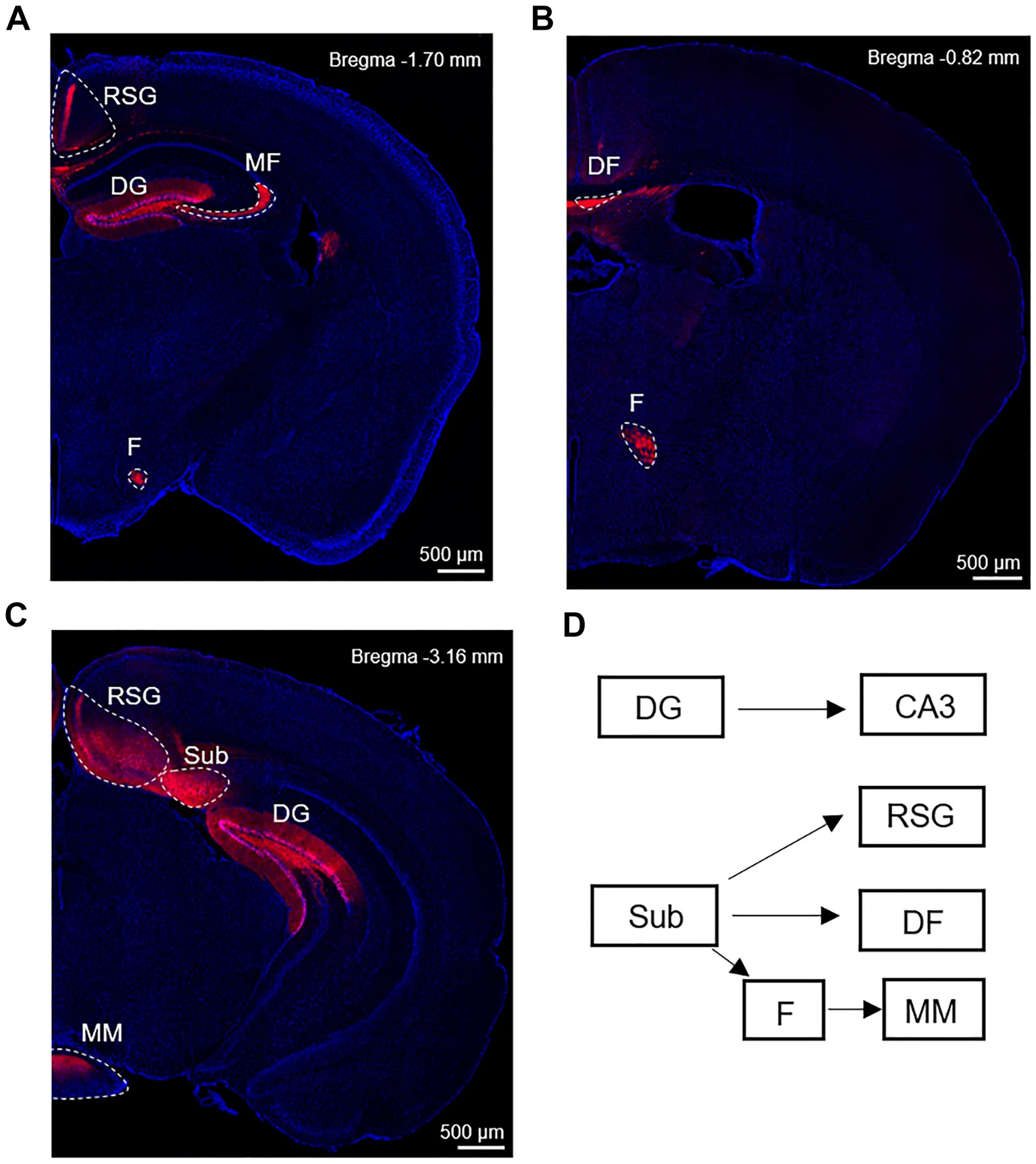
Axon projections of Nrg1-positive neurons in the hippocampus. **A–C** Expression of tdTomato in coronal brain slices at the positions of bregma − 1.7 mm **A**, − 0.82 mm **B** and − 3.16 mm **C** after injection of Cre-reporting AAV into the DG and Sub of 2-month-old Nrg1^Cre/+^ mice. Four weeks after AAV injection, the brain slices were collected and analyzed. Scale bar, 500 μm. **D** Summary of the projections of Nrg1-positive neurons in the DG and Sub of mouse hippocampus. DG: dentate gyrus; RSG: retrosplenial granular cortex; MF: mossy fiber; F: fornix; DF: dorsal fornix; Sub: subiculum; MM: mammillary nucleus

We further analyzed the tdTomato expression in a series of coronal sections to explore the projections of Nrg1-positive cells in adult mouse hippocampus. We found tdTomato expression in the cells of DG and mossy fiber of CA3 region (Fig. 6A). Moreover, tdTomato was expressed in the cells of Sub and axon fibers in the retrosplenial granular cortex (RSG), dorsal fornix (DF), fornix (F) and mammillary nucleus (MM) (Fig. 6A–C). Intriguingly, Sub pyramidal neurons send their axon projections to MM through fornix, which is part of the Papez circuit [43]. Taken together, these results demonstrate that Nrg1 is mainly expressed in DG-CA3 pathway and Papez circuit in mouse hippocampus (Fig. 6D).

### Expression pattern of Nrg1 in the hypothalamus and thalamus

**Fig. 7:**
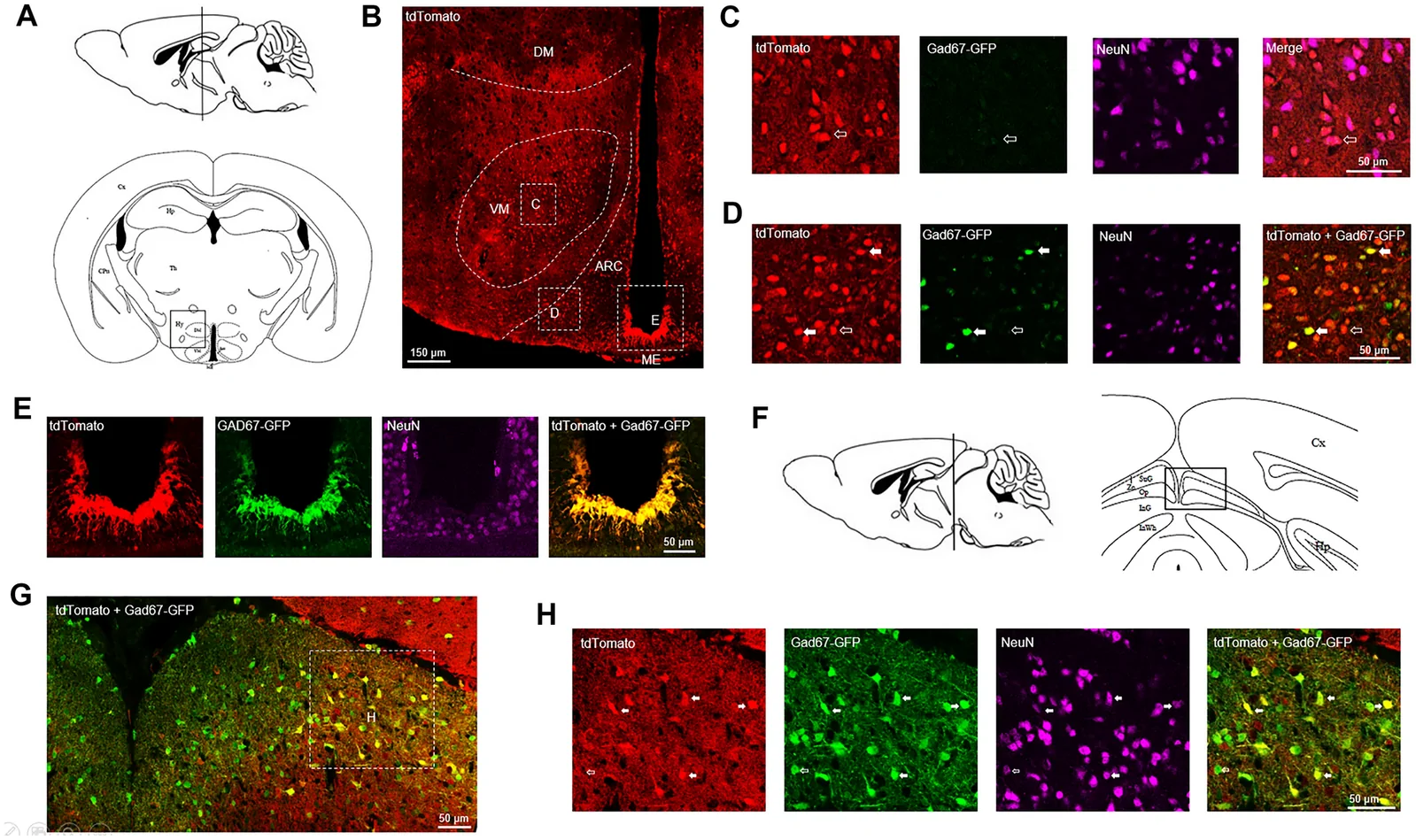
Expression pattern of Nrg1 in the hypothalamus and thalamus. **A** Diagram of mouse brain sagittal section (top) and coronal section (bottom). The line in the sagittal section diagram indicates the position of the coronal section. The rectangle indicates the brain region shown in panel B. **B** Expression of tdTomato in the hypothalamus of 2-month-old Nrg1-reporting mice. Scale bar, 150 μm. **C–E** Fluorescent images of tdTomato, Gad67-GFP and NeuN in the VM **C**, ARC **D** and ME **E** from the rectangles in panel B. The solid arrows indicate Nrg1-positive cells which are GABAergic. The empty arrows indicate Nrg1-positive cells not expressing Gad67-GFP. Scale bars, 50 μm. The Nrg1-reporting mice were crossed with Gad67-GFP mice to visualize the expression of Nrg1 in GABAergic neurons. **F** Diagram of mouse brain sagittal section (left) and coronal section (right). The line in the sagittal section diagram indicates the position of the coronal section. The rectangle indicates the brain region shown in panel G. **G** Expression of tdTomato and Gad67-GFP in the SC of thalamus from 2-month-old Nrg1-reporting mice. Scale bar, 50 μm. **H** Fluorescent images of tdTomato, Gad67-GFP and NeuN from the rectangle in panel G. The solid arrows indicate GABAergic neurons expressing Nrg1. The empty arrow indicates GABAergic neurons not expressing Nrg1. Scale bar, 50 μm. DM: dorsomedial nucleus; VM: ventromedial nucleus; ARC: arcuate nucleus; ME: median eminence

The hypothalamus is a deep structure in the brain and is localized below the thalamus (Fig. 7A). The hypothalamus is important for regulating metabolism, temperature and instinctive behaviors such as sleep, hunger, thirst, fear and maternal behaviors. In the hypothalamus of Nrg1-reporting mice, the tdTomato-positive cells were mainly distributed in the ventromedial nucleus (VM), arcuate nucleus (ARC) and median eminence (ME) (Fig. 7B). These results suggest that Nrg1 is mostly expressed in the VM, Arc and ME of mouse hypothalamus. We also crossed Nrg1-reporting mice with Gad67-GFP mice to see whether Nrg1 was expressed in GABAergic interneurons. In the VM, Nrg1 is expressed in putative excitatory neurons as tdTomato is co-localized with NeuN (a pan neuronal marker) but not GFP (Fig. 7C).

The ARC contains both NPY/AgRP neurons, which are inhibited by insulin and leptin and, when activated, stimulated food intake, and POMC neurons, which reduce food intake and are stimulated by insulin and leptin [44]. NPY/AgRP neurons can inhibit POMC neurons via synaptic release of GABA [45]. Interestingly, about two-third of tdTomato-positive cells are GABAergic in the ARC (Fig. 7D), indicating this population of Nrg1-positive cells are probably AgRP neurons. The tdTomato signal is highly co-localized with GFP in the ME (Fig. 7E), a region that severs as the gateway for release of hypothalamic hormones [46]. In the thalamus, the tdTomato-positive cells are mainly distributed in the superior colliculus (SC) (Fig. 7F, G), a structure that is involved in coordinating eye and head movements [47]. These results suggest that Nrg1 is highly expressed in the SC of mouse thalamus. Intriguingly, nearly all tdTomato-positive cells in the SC express GFP but not vice versa (Fig. 7H). These results suggest that Nrg1 is mostly expressed in GABAergic interneurons, but not all GABAergic interneurons express Nrg1 in the mouse SC.

### Expression pattern of Nrg1 in the cerebellum

The cerebellum plays important roles in motor control, emotion and cognitive functions such as attention and language. The cerebellar cortex is divided into three layers. At the bottom lies the thick granular layer (GL), densely packed with granule cells, along with interneurons. In the middle lies the Purkinje layer (PL), a narrow zone that contains the cell bodies of Purkinje cells and Bergmann glia (BG) cells. At the top lies the molecular layer (ML), which contains the dendritic trees of Purkinje cells, along with two types of GABAergic interneurons: stellate cells and basket cells.

**Fig. 8:**
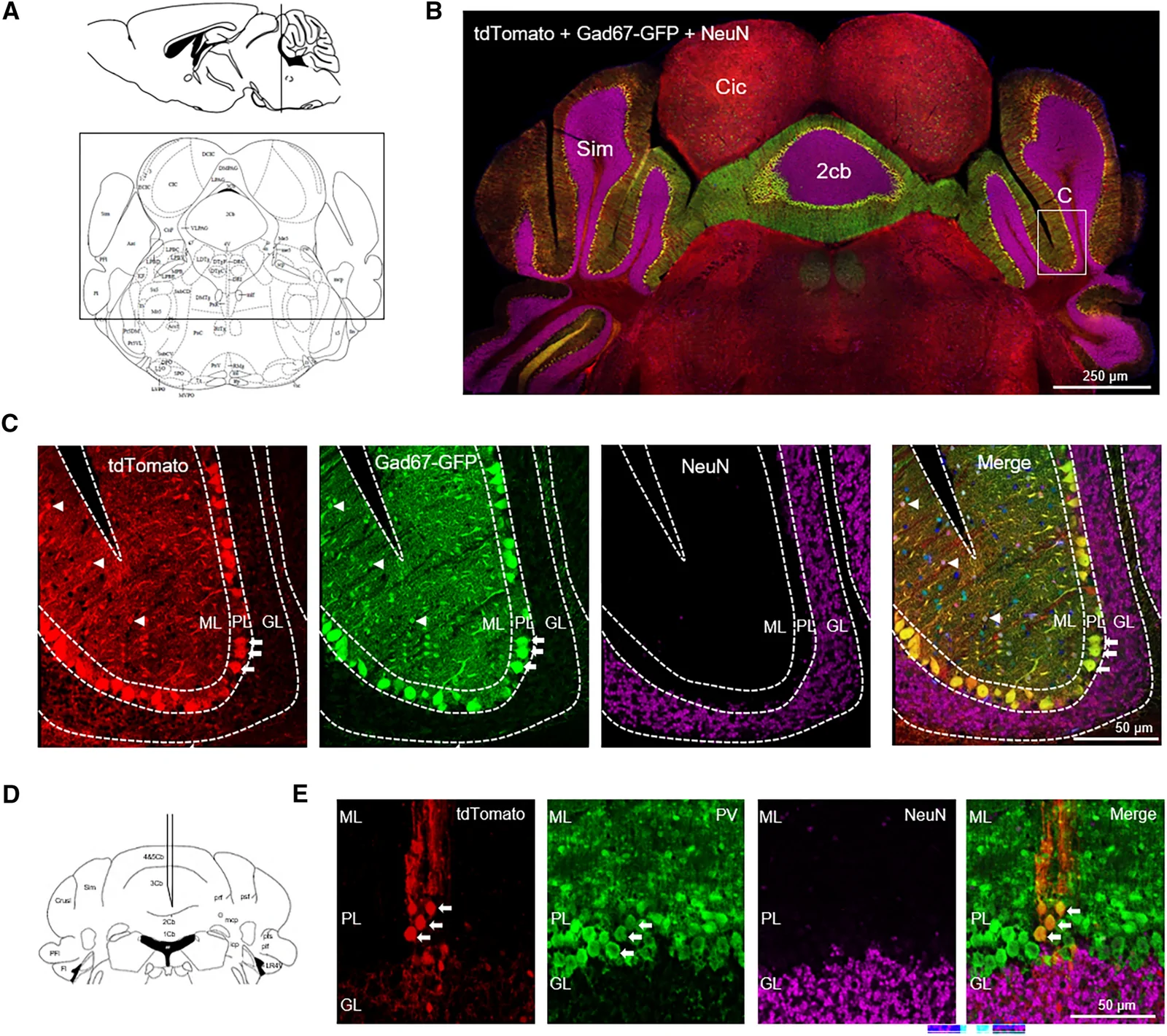
Expression pattern of Nrg1 in the cerebellum. **A** Diagram of mouse brain sagittal section (top) and coronal section (bottom). The line in the sagittal section diagram indicates the position of the coronal section. The rectangle indicates the brain region shown in panel B. **B** Fluorescent images of tdTomato, Gad67-GFP and NeuN in the cerebellum of 2-month-old Nrg1-reporting mice. Scale bar, 250 μm. **C** Fluorescent images of tdTomato, Gad67-GFP and NeuN in three layers of cerebellar cortex from the rectangle in panel B. The arrows indicate the Nrg1-positive Purkinje cells, and the arrowheads indicate the Nrg1-expressing GABAergic interneurons. Scale bar, 50 μm. The Nrg1-reporting mice were crossed with Gad67-GFP mice to visualize the expression of Nrg1 in GABAergic interneurons. **D** Diagram showing the stereotaxic injection of Cre-reporting AAV into the PL of 2-month-old Nrg1^Cre/+^ mice. Four weeks after AAV injection, the brain slices were collected and analyzed. **E** Immunofluorescent images of tdTomato and PV in the PL of SC of Nrg1^Cre/+^ mice. The arrows indicate Purkinje cells expressing Nrg1 in adult mouse cerebellum. Scale bar, 50 μm. Sim: simple lobule; Cic: commissure of the inferior colliculus; 2cb: 2nd cerebellar lobule; GL: granular layer; PL: Purkinje layer; ML: molecular layer; PV: parvalbumin

In the Nrg1-reporting mice, we found that tdTomato is mainly expressed in the PL and ML, but absent in the GL (Fig. 8A, B). We crossed the Nrg1-reporting mice with Gad67-GFP mice to study whether Nrg1 is expressed in the GABAergic interneurons. The tdTomato proteins were mainly expressed in the cell body of Purkinje cells in the PL, as well as in the dendritic trees of Purkinje cells and some GABAergic interneurons in the ML (Fig. 8C). Although NeuN is present in most neuronal cell types, it is not expressed in Purkinje cells (Fig. 8C), consistent with previous findings [48]. To verify that Nrg1 is expressed in the Purkinje cells in adult mice, we injected Cre-reporting AAV into the PL of cerebellum in 2-month-old Nrg1^Cre/+^ mice (Fig. 8D). As shown in Fig. 8E, the tdTomato proteins were expressed in the cell bodies and dendritic trees of Purkinje cells, indicated by the co-localization of tdTomato with parvalbumin (PV), a protein marker for Purkinje cells. These results solidify the finding from Nrg1-reporting mice that Nrg1 is indeed expressed in the Purkinje cells of adult mice.

## Discussion

In this paper, we generated Nrg1-reporting mice using the cre-loxp strategy to investigate the cellular expression pattern of Nrg1 in mouse brain. Meanwhile we revealed the axon projections of Nrg1-positive neurons in the mouse basal ganglion and hippocampus. These results provide fundamental information needed for understanding the function of NRG1 at the cellular and circuit levels. Here, we discuss the findings revealed by Nrg1-reporting mice and their relevance to physiology and brain disorders.

The results from Nrg1-reporting mice indicated that Nrg1 was highly expressed in the granule cells of mouse OB. These findings are consistent with the previous study showing Nrg1 expression in the developing mouse OB [49]. The reciprocal dendro-dendritic synapses formed between mitral and granule cells are considered to be important for the synchronization of mitral cells, affecting the ability of odor discrimination [50]. Intriguingly, a recent study indicated that the ErbB4 receptor of NRG1 is selectively expressed in the GABAergic interneurons in the GL and GCL of OB and regulates the ability of ordor discrimination in mice [51]. NRG1-ErbB4 has been implicated in regulating synapse formation or function through a cell-adhesion and diffusion manner, respectively [12, 14, 18, 52]. Future works are warranted to study the role of NRG1-ErbB4 signaling in the formation and function of synapse between mitral and granule cell, and whether disruption of NRG1-ErbB4 signaling in the OB causes olfactory dysfunction that is commonly observed in schizophrenia patients [53].

The data from Nrg1-reporting mice revealed that Nrg1 was mainly expressed in the layer 2–3 of cerebral cortex. By contrast, a previous study using in situ hybridization showed that type I/II Nrg1 mRNA was mainly expressed in layer 2–3 while type III Nrg1 mRNA was mostly expressed in layer 5 of mouse cerebral cortex [14]. These discrepancies could be due to the different sensitivity to reflect the expression of different Nrg1 splicing isoforms between the methods of genetic reporter and in situ hybridization. The pyramidal neurons in the layer 2–3 of cerebral cortex are responsible for the communication between different cortical regions [54, 55]. Future work is required to investigate the function of Nrg1 in regulating the crosstalk between different cortical regions.

Here we showed that Nrg1 was highly expressed in the Drd1-positive MSNs of mouse striatum, which is consistent with the single-cell RNA sequencing results from mouse striatum [29]. We further showed that Nrg1-positive neurons in the NAc send their axon projections the SNr and thus were in the direct pathway of basal ganglion. Interestingly, the ErbB4 receptor is expressed in the dopaminergic neurons in the SNr [31, 56]. The dysfunction of the direct pathway in the basal ganglion leads to deficits in locomotion and sociability [57, 58]. Future study is warranted to investigate how the dysregulation of NRG1-ErbB4 signaling in the NAc would affect the balance between direct and indirect pathway in the basal ganglion.

The expression of Nrg1 in the DG of mouse hippocampus has been reported by previous studies [14, 59], which is in line with our findings in the Nrg1-reporting mice. Although previous studies showed moderate expression of Nrg1 in the CA1-3 pyramidal neurons [14, 26], the results from Nrg1-reporting mice indicate sparse expression of Nrg1 in the CA1-3 regions of hippocampus. By contrast, we showed here that Nrg1 is highly expressed in the subiculum, the major output region of hippocampus. The subiculum has projections to many cortical and subcortical regions. Here we showed that Nrg1-positive neurons in the subiculum send axon projections to RSG where the Erbb4 receptor is also highly expressed [31]. Moreover, Nrg1-positive neurons in the subiculum also have projections to the mammillary nucleus through fornix, which belongs to the Papez circuit [43]. It will be interesting to study the function of Nrg1 in the Papez circuit that plays important roles in the regulation of emotion [60, 61]. The data from Nrg1-reporting mice indicated that Nrg1 was mainly expressed in neurons but not astrocytes in the hippocampus under basal conditions. However, it is possible that Nrg1 can be expressed in reactive astrocytes in vivo or primary cultured astrocytes in vitro [26, 27].

The functions of Nrg1 in the thalamus and hypothalamus are relatively less well understood compared to cerebral cortex and hippocampus. The finding here that Nrg1 is highly expressed in the SC subregion of thalamus raises a possibility for Nrg1 in regulating coordination between visual stimulation and movement. We found here that Nrg1 is highly expressed in the ARC of hypothalamus. The ARC has no blood–brain barrier and can be directly affected by the circulating hormones such as insulin and leptin. Our results indicate that Nrg1 might be expressed in both the NPY/AgRP neurons and POMC neurons. Both types of neurons in the ARC project to adjacent paraventricular nucleus (PVN) to reduce food intake. Intriguingly, the ErbB4 receptor of NRG1 is highly expressed in the PVN of hypothalamus [31]. These results suggest the possibility that NRG1-ErbB4 signaling might regulate food intake through the ARC-PVH circuit. Nrg1 is also highly expressed in the GABAergic interneurons of ME, which raises a possibility that Nrg1 may regulate the release of hypothalamus hormones.

Here we found that Nrg1 is strongly expressed in the Purkinje cells in the PL of cerebellum. By contrast, the ErbB4 receptor of NRG1 is not expressed in the PL of cerebellum [31]. However, the ErbB3 receptor is highly expressed in the Bergmann glia cells in the PL and important for BG development and cerebellar lamination [62]. These results indicate that NRG1-ErbB3 signaling might regulate the maturation of BG cells. Nrg1 is also expressed in the GABAergic interneurons in the GL of cerebellum. In contrast, the ErbB4 receptor is expressed in a few glia cells but not GABAergic interneurons in the GL [31]. Future studies are warranted to investigate the role of Nrg1 in regulating the cerebellum functions.

## Conclusion

Nrg1 is broadly expressed in mouse brain, mainly in neurons, but has unique expression patterns in different brain regions. The Nrg1-expressing neurons are highly presented in the direct pathway of basal ganglia, the DG to CA3 pathway and the Papez circuit. The data presented here may provide fundamental information for the study of the functions of Nrg1 and the related neural circuits in the future.

## Supplementary Information

**Additional file 1: Figure S1.** The densities of Nrg1-positive cells in different brain regions of adult Nrg1-reporting mice. n = 3.

**Additional file 2: Figure S2.** Nrg1 is not expressed in astrocytes in adult mouse hippocampus. The dentate gyrusof Nrg1-reporting mice were subjected to immunostaining with anti-GFAPor anti-S100βantibodies. Scale bar, 25μm.

## Data Availability

All data generated during this study are included in this published article. All unique materials used in this study are available from the corresponding author on reasonable request.

## Abbreviations

Nrg1: Neuregulin 1
AAV: Adeno-associated virus
OB: Olfactory bulb
PG: Periglomerular
NAc: Nucleus accumbens
SNr: Substantia nigra pars reticulata
RSG: Retrosplenial granular cortex
MM: Mammillary nucleus
ECD: Extracellular domain
ICD: Intracellular domain
LTP: Long-term potentiation
LIMK1: LIM kinase 1
FISH: Fluorescence in situ hybridization
TRAP: Translating ribosome affinity purification
WT: Wild type
ONL: Olfactory nerve layer
GL: Glomerular layer
EPL: External plexiform layer
ML: Mitral layer
IPL: Internal plexiform layer
GCL: Granular cell layer
RMS: Rostral migratory stream
Cg1: Cingulate cortex, area 1
RS: Retrosplenial cortex
Pir: Piriform cortex
Au: Auditory cortex
M1: Primary motor cortex
M2: Secondary motor cortex
SS1: Primary somatosensory cortex
SS2: Secondary somatosensory cortex
LE: Lateral entorhinal cortex
PrL: Prelimibic cortex
CPu: Caudate putamen
MSN: Medium spiny neurons
SN: Substantia nigra
Tu: Olfactory tubercle
Drd1: Dopamine D1 receptor
Drd2: Dopamine D2 receptor
GPe: External globus pallidus
CA: Cornu ammonis
DG: Dentate gyrus
Sub: Subiculum
FC: Fasciola cinereum
GFAP: Glial fibrillary acidic protein
DF: Dorsal fornix
F: Fornix
MM: Mammillary nucleus
DM: Dorsomedial nucleus
VM: Ventromedial nucleus
ARC: Arcuate nucleus
ME: Median eminence
AgRP: Agouti-related protein
NPY: Neuropeptide Y
POMC: Pro-opiomelanocortin
SC: Superior colliculus
Sim: Simple lobule
Cic: Commissure of the inferior colliculus
2cb: 2Nd cerebellar lobule
GL: Granular layer
PL: Purkinje layer
ML: Molecular layer
PV: Parvalbumin
BG: Bergmann glia

## References

[1] Kato T. Molecular genetics of bipolar disorder and depression. Psychiatry Clin Neurosci. 2007;61(1):3–19. 10.1111/j.1440-1819.2007.01604.x 17239033

[2] Stefansson H, Sigurdsson E, Steinthorsdottir V, Bjornsdottir S, Sigmundsson T, Ghosh S, Brynjolfsson J, Gunnarsdottir S, Ivarsson O, Chou TT, et al. Neuregulin 1 and susceptibility to schizophrenia. Am J Hum Genet. 2002;71(4):877–92. 10.1086/342734 PMC378543 12145742

[3] Yang JZ, Si TM, Ruan Y, Ling YS, Han YH, Wang XL, Zhou M, Zhang HY, Kong QM, Liu C, et al. Association study of neuregulin 1 gene with schizophrenia. Mol Psychiatry. 2003;8(7):706–9. 10.1038/sj.mp.4001377 12874607

[4] Mei L, Nave K-A. Neuregulin-ERBB signaling in the nervous system and neuropsychiatric diseases. Neuron. 2014;83(1):27–49. 10.1016/j.neuron.2014.06.007 PMC4189115 24991953

[5] Xu Z, Jiang J, Ford G, Ford BD. Neuregulin-1 is neuroprotective and attenuates inflammatory responses induced by ischemic stroke. Biochem Biophys Res Commun. 2004;322(2):440–6. 10.1016/j.bbrc.2004.07.149 15325249

[6] Tan G-H, Liu Y-Y, Hu X-L, Yin D-M, Mei L, Xiong Z-Q. Neuregulin 1 represses limbic epileptogenesis through ErbB4 in parvalbumin-expressing interneurons. Nat Neurosci. 2011;15(2):258–66. 10.1038/nn.3005 22158510

[7] Ryu J, Hong BH, Kim YJ, Yang EJ, Choi M, Kim H, Ahn S, Baik TK, Woo RS, Kim HS. Neuregulin-1 attenuates cognitive function impairments in a transgenic mouse model of Alzheimer’s disease. Cell Death Dis. 2016;7(2):e2117. 10.1038/cddis.2016.30 PMC4849157 26913607

[8] Li K-X, Lu Y-M, Xu Z-H, Zhang J, Zhu J-M, Zhang J-M, Cao S-X, Chen X-J, Chen Z, Luo J-H, et al. Neuregulin 1 regulates excitability of fast-spiking neurons through Kv11 and acts in epilepsy. Nat Neurosci. 2011;15(2):267–73. 10.1038/nn.3006 22158511

[9] Mei L, Xiong W-C. Neuregulin 1 in neural development, synaptic plasticity and schizophrenia. Nat Rev Neurosci. 2008;9(6):437–52. 10.1038/nrn2392 PMC2682371 18478032

[10] Brinkmann BG, Agarwal A, Sereda MW, Garratt AN, Müller T, Wende H, Stassart RM, Nawaz S, Humml C, Velanac V, et al. Neuregulin-1/ErbB signaling serves distinct functions in myelination of the peripheral and central nervous system. Neuron. 2008;59(4):581–95. 10.1016/j.neuron.2008.06.028 PMC2628490 18760695

[11] Del Pino I, García-Frigola C, Dehorter N, Brotons-Mas JR, Alvarez-Salvado E, Martínez de Lagrán M, Ciceri G, Gabaldón MV, Moratal D, Dierssen M, et al. Erbb4 deletion from fast-spiking interneurons causes schizophrenia-like phenotypes. Neuron. 2013;79(6):1152–68. 10.1016/j.neuron.2013.07.010 24050403

[12] Fazzari P, Paternain AV, Valiente M, Pla R, Luján R, Lloyd K, Lerma J, Marín O, Rico B. Control of cortical GABA circuitry development by Nrg1 and ErbB4 signalling. Nature. 2010;464(7293):1376–80. 10.1038/nature08928 20393464

[13] Ting AK, Chen Y, Wen L, Yin D-M, Shen C, Tao Y, Liu X, Xiong W-C, Mei L. Neuregulin 1 promotes excitatory synapse development and function in GABAergic interneurons. J Neurosci. 2011;31(1):15–25. 10.1523/JNEUROSCI.2538-10.2011 PMC3078582 21209185

[14] Woo R-S, Li X-M, Tao Y, Carpenter-Hyland E, Huang YZ, Weber J, Neiswender H, Dong X-P, Wu J, Gassmann M, et al. Neuregulin-1 enhances depolarization-induced GABA release. Neuron. 2007;54(4):599–610. 10.1016/j.neuron.2007.04.009 17521572

[15] Wen L, Lu Y-S, Zhu X-H, Li X-M, Woo R-S, Chen Y-J, Yin D-M, Lai C, Terry AV, Vazdarjanova A, et al. Neuregulin 1 regulates pyramidal neuron activity via ErbB4 in parvalbumin-positive interneurons. Proc Natl Acad Sci U S A. 2010;107(3):1211–6. 10.1073/pnas.0910302107 PMC2824309 20080551

[16] Chen Y-J, Zhang M, Yin D-M, Wen L, Ting A, Wang P, Lu Y-S, Zhu X-H, Li S-J, Wu C-Y, et al. ErbB4 in parvalbumin-positive interneurons is critical for neuregulin 1 regulation of long-term potentiation. Proc Natl Acad Sci U S A. 2010;107(50):21818–23. 10.1073/pnas.1010669107 PMC3003111 21106764

[17] Tan Z, Robinson HL, Yin DM, Liu Y, Liu F, Wang H, Lin TW, Xing G, Gan L, Xiong WC, et al. Dynamic ErbB4 activity in hippocampal-prefrontal synchrony and top-down attention in rodents. Neuron. 2018;98(2):380–93. 10.1016/j.neuron.2018.03.018 PMC5909841 29628188

[18] Huang YZ, Won S, Ali DW, Wang Q, Tanowitz M, Du QS, Pelkey KA, Yang DJ, Xiong WC, Salter MW, et al. Regulation of neuregulin signaling by PSD-95 interacting with ErbB4 at CNS synapses. Neuron. 2000;26(2):443–55. 10.1016/s0896-6273(00)81176-9 10839362

[19] Chen P, Jing H, Xiong M, Zhang Q, Lin D, Ren D, Wang S, Yin D, Chen Y, Zhou T, et al. Spine impairment in mice high-expressing neuregulin 1 due to LIMK1 activation. Cell Death Dis. 2021;12(4):403. 10.1038/s41419-021-03687-8 PMC8047019 33854034

[20] Wang Y-Y, Zhao B, Wu M-M, Zheng X-L, Lin L, Yin D-M. Overexpression of neuregulin 1 in GABAergic interneurons results in reversible cortical disinhibition. Nat Commun. 2021;12(1):278. 10.1038/s41467-020-20552-y PMC7804852 33436636

[21] Wang JY, Miller SJ, Falls DL. The N-terminal region of neuregulin isoforms determines the accumulation of cell surface and released neuregulin ectodomain. J Biol Chem. 2001;276(4):2841–51. 10.1074/jbc.M005700200 11042203

[22] Yin D-M, Chen Y-J, Lu Y-S, Bean JC, Sathyamurthy A, Shen C, Liu X, Lin TW, Smith CA, Xiong W-C, et al. Reversal of behavioral deficits and synaptic dysfunction in mice overexpressing neuregulin 1. Neuron. 2013;78(4):644–57. 10.1016/j.neuron.2013.03.028 PMC4176764 23719163

[23] Bao J, Wolpowitz D, Role LW, Talmage DA. Back signaling by the Nrg-1 intracellular domain. J Cell Biol. 2003;161(6):1133–41. 10.1083/jcb.200212085 PMC2172983 12821646

[24] Bao J, Lin H, Ouyang Y, Lei D, Osman A, Kim T-W, Mei L, Dai P, Ohlemiller KK, Ambron RT. Activity-dependent transcription regulation of PSD-95 by neuregulin-1 and Eos. Nat Neurosci. 2004;7(11):1250–8. 10.1038/nn1342 15494726

[25] Navarro-González C, Huerga-Gómez A, Fazzari P. Nrg1 intracellular signaling is neuroprotective upon stroke. Oxid Med Cell Longev. 2019;2019:3930186. 10.1155/2019/3930186 PMC6754950 31583038

[26] Kerber G, Streif R, Schwaiger F-W, Kreutzberg GW, Hager G. Neuregulin-1 isoforms are differentially expressed in the intact and regenerating adult rat nervous system. J Mol Neurosci. 2003;21(2):149–65. 10.1385/JMN:21:2:149 14593214

[27] Liu X, Bates R, Yin D-M, Shen C, Wang F, Su N, Kirov SA, Luo Y, Wang J-Z, Xiong W-C, et al. Specific regulation of NRG1 isoform expression by neuronal activity. J Neurosci. 2011;31(23):8491–501. 10.1523/JNEUROSCI.5317-10.2011 PMC3154699 21653853

[28] Sun Y, Ikrar T, Davis MF, Gong N, Zheng X, Luo ZD, Lai C, Mei L, Holmes TC, Gandhi SP, et al. Neuregulin-1/ErbB4 signaling regulates visual cortical plasticity. Neuron. 2016;92(1):160–73. 10.1016/j.neuron.2016.08.033 PMC5310354 27641496

[29] Saunders A, Macosko EZ, Wysoker A, Goldman M, Krienen FM, de Rivera H, Bien E, Baum M, Bortolin L, Wang S, et al. Molecular diversity and specializations among the cells of the adult mouse brain. Cell. 2018. 10.1016/j.cell.2018.07.028. PMC6447408 30096299

[30] Vullhorst D, Neddens J, Karavanova I, Tricoire L, Petralia RS, McBain CJ, Buonanno A. Selective expression of ErbB4 in interneurons, but not pyramidal cells, of the rodent hippocampus. J Neurosci. 2009;29(39):12255–64. 10.1523/JNEUROSCI.2454-09.2009 PMC2774835 19793984

[31] Bean JC, Lin TW, Sathyamurthy A, Liu F, Yin D-M, Xiong W-C, Mei L. Genetic labeling reveals novel cellular targets of schizophrenia susceptibility gene: distribution of GABA and non-GABA ErbB4-positive cells in adult mouse brain. J Neurosci. 2014;34(40):13549–66. 10.1523/JNEUROSCI.2021-14.2014 PMC4180480 25274830

[32] Agarwal A, Zhang M, Trembak-Duff I, Unterbarnscheidt T, Radyushkin K, Dibaj P, Martins de Souza D, Boretius S, Brzózka MM, Steffens H, et al. Dysregulated expression of neuregulin-1 by cortical pyramidal neurons disrupts synaptic plasticity. Cell Rep. 2014;8(4):1130–45. 10.1016/j.celrep.2014.07.026 25131210

[33] Navarro-Gonzalez C, Carceller H, Benito Vicente M, Serra I, Navarrete M, Domínguez-Canterla Y, Rodríguez-Prieto Á, González-Manteiga A, Fazzari P. Nrg1 haploinsufficiency alters inhibitory cortical circuits. Neurobiol Dis. 2021;157:105442. 10.1016/j.nbd.2021.105442 34246770

[34] Law AJ, Shannon Weickert C, Hyde TM, Kleinman JE, Harrison PJ. Neuregulin-1 (NRG-1) mRNA and protein in the adult human brain. Neuroscience. 2004;127(1):125–36. 10.1016/j.neuroscience.2004.04.026 15219675

[35] Madisen L, Zwingman TA, Sunkin SM, Oh SW, Zariwala HA, Gu H, Ng LL, Palmiter RD, Hawrylycz MJ, Jones AR, et al. A robust and high-throughput Cre reporting and characterization system for the whole mouse brain. Nat Neurosci. 2010;13(1):133–40. 10.1038/nn.2467 PMC2840225 20023653

[36] Yu Q, Liu Y-Z, Zhu Y-B, Wang Y-Y, Li Q, Yin D-M. Genetic labeling reveals temporal and spatial expression pattern of D2 dopamine receptor in rat forebrain. Brain Struct Funct. 2019;224(3):1035–49. 10.1007/s00429-018-01824-2 PMC6499762 30604007

[37] Zhang Y-Q, Lin W-P, Huang L-P, Zhao B, Zhang C-C, Yin D-M. Dopamine D2 receptor regulates cortical synaptic pruning in rodents. Nat Commun. 2021;12(1):6444. 10.1038/s41467-021-26769-9 PMC8576001 34750364

[38] Kim JH, Lee SR, Li LH, Park HJ, Park JH, Lee KY, Kim MK, Shin BA, Choi SY. High cleavage efficiency of a 2A peptide derived from porcine teschovirus-1 in human cell lines, zebrafish and mice. PLoS ONE. 2011;6(4):e18556. 10.1371/journal.pone.0018556 PMC3084703 21602908

[39] Li D, Qiu Z, Shao Y, Chen Y, Guan Y, Liu M, Li Y, Gao N, Wang L, Lu X, et al. Heritable gene targeting in the mouse and rat using a CRISPR-Cas system. Nat Biotechnol. 2013;31(8):681–3. 10.1038/nbt.2661 23929336

[40] Tamamaki N, Yanagawa Y, Tomioka R, Miyazaki J-I, Obata K, Kaneko T. Green fluorescent protein expression and colocalization with calretinin, parvalbumin, and somatostatin in the GAD67-GFP knock-in mouse. J Comp Neurol. 2003;467(1):60–79. 10.1002/cne.10905 14574680

[41] Crittenden JR, Gipson TA, Smith AC, Bowden HA, Yildirim F, Fischer KB, Yim M, Housman DE, Graybiel AM. Striatal transcriptome changes linked to drug-induced repetitive behaviors. Eur J Neurosci. 2021;53(8):2450–68. 10.1111/ejn.15116 PMC8330602 33759265

[42] Gerfen CR, Surmeier DJ. Modulation of striatal projection systems by dopamine. Annu Rev Neurosci. 2011;34:441–66. 10.1146/annurev-neuro-061010-113641 PMC3487690 21469956

[43] Shah A, Jhawar SS, Goel A. Analysis of the anatomy of the Papez circuit and adjoining limbic system by fiber dissection techniques. J Clin Neurosci. 2012;19(2):289–98. 10.1016/j.jocn.2011.04.039 22209397

[44] Morton GJ, Cummings DE, Baskin DG, Barsh GS, Schwartz MW. Central nervous system control of food intake and body weight. Nature. 2006;443(7109):289–95. 10.1038/nature05026 16988703

[45] Cowley MA, Smart JL, Rubinstein M, Cerdan MG, Diano S, Horvath TL, Cone RD, Low MJ. Leptin activates anorexigenic POMC neurons through a neural network in the arcuate nucleus. Nature. 2001;411(6836):480–4. 10.1038/35078085 11373681

[46] Palkovits M. Neuropeptides in the hypothalamo-hypophyseal system: lateral retrochiasmatic area as a common gate for neuronal fibers towards the median eminence. Peptides. 1984;5(Suppl 1):35–9. 10.1016/0196-9781(84)90262-6 6148739

[47] Gandhi NJ, Katnani HA. Motor functions of the superior colliculus. Annu Rev Neurosci. 2011;34:205–31. 10.1146/annurev-neuro-061010-113728 PMC3641825 21456962

[48] Wolf HK, Buslei R, Schmidt-Kastner R, Schmidt-Kastner PK, Pietsch T, Wiestler OD, Blumcke I. NeuN: a useful neuronal marker for diagnostic histopathology. J Histochem Cytochem. 1996;44(10):1167–71. 10.1177/44.10.8813082 8813082

[49] Bovetti S, De Marchis S, Gambarotta G, Fasolo A, Perroteau I, Puche AC, Bovolin P. Differential expression of neuregulins and their receptors in the olfactory bulb layers of the developing mouse. Brain Res. 2006;1077(1):37–47. 10.1016/j.brainres.2006.01.034 16488402

[50] Schoppa NE. Synchronization of olfactory bulb mitral cells by precisely timed inhibitory inputs. Neuron. 2006;49(2):271–83. 10.1016/j.neuron.2005.11.038 16423700

[51] Tan Z, Liu Z, Liu Y, Liu F, Robinson H, Lin TW, Xiong WC, Mei L. An ErbB4-positive neuronal network in the olfactory bulb for olfaction. J Neurosci. 2022;42(34):6518–35. 10.1523/JNEUROSCI.0131-22.2022 PMC9410760 35853717

[52] Krivosheya D, Tapia L, Levinson JN, Huang K, Kang Y, Hines R, Ting AK, Craig AM, Mei L, Bamji SX, et al. ErbB4-neuregulin signaling modulates synapse development and dendritic arborization through distinct mechanisms. J Biol Chem. 2008;283(47):32944–56. 10.1074/jbc.M800073200 PMC5395083 18819924

[53] Moberg PJ, Agrin R, Gur RE, Gur RC, Turetsky BI, Doty RL. Olfactory dysfunction in schizophrenia: a qualitative and quantitative review. Neuropsychopharmacology. 1999;21(3):325–40. 10.1016/S0893-133X(99)00019-6 10457530

[54] Feldmeyer D, Lübke J, Sakmann B. Efficacy and connectivity of intracolumnar pairs of layer 2/3 pyramidal cells in the barrel cortex of juvenile rats. J Physiol. 2006;575(Pt 2):583–602. 10.1113/jphysiol.2006.105106 PMC1819447 16793907

[55] Wozny C, Williams SR. Specificity of synaptic connectivity between layer 1 inhibitory interneurons and layer 2/3 pyramidal neurons in the rat neocortex. Cereb Cortex. 2011;21(8):1818–26. 10.1093/cercor/bhq257 PMC3138515 21220765

[56] Zheng Y, Watakabe A, Takada M, Kakita A, Namba H, Takahashi H, Yamamori T, Nawa H. Expression of ErbB4 in substantia nigra dopamine neurons of monkeys and humans. Prog Neuropsychopharmacol Biol Psychiatry. 2009;33(4):701–6. 10.1016/j.pnpbp.2009.03.021 19336245

[57] Shonesy BC, Parrish WP, Haddad HK, Stephenson JR, Báldi R, Bluett RJ, Marks CR, Centanni SW, Folkes OM, Spiess K, et al. Role of striatal direct pathway 2-arachidonoylglycerol signaling in sociability and repetitive behavior. Biol Psychiatry. 2018;84(4):304–15. 10.1016/j.biopsych.2017.11.036 PMC6023784 29458998

[58] Aristieta A, Barresi M, Azizpour Lindi S, Barrière G, Courtand G, de la Crompe B, Guilhemsang L, Gauthier S, Fioramonti S, Baufreton J, et al. A disynaptic circuit in the globus pallidus controls locomotion inhibition. Curr Biol. 2021. 10.1016/j.cub.2020.11.019. 33306949

[59] Mahar I, Tan S, Davoli MA, Dominguez-Lopez S, Qiang C, Rachalski A, Turecki G, Mechawar N. Subchronic peripheral neuregulin-1 increases ventral hippocampal neurogenesis and induces antidepressant-like effects. PLoS ONE. 2011;6(10):e26610. 10.1371/journal.pone.0026610 PMC3197569 22028923

[60] Pessoa L, McMenamin B. Dynamic networks in the emotional brain. Neuroscientist. 2017;23(4):383–96. 10.1177/1073858416671936 PMC5405011 27784761

[61] Pratt JA. The neuroanatomical basis of anxiety. Pharmacol Ther. 1992;55(2):149–81. 10.1016/0163-7258(92)90014-q 1363249

[62] Sathyamurthy A, Yin DM, Barik A, Shen C, Bean JC, Figueiredo D, She JX, Xiong WC, Mei L. ERBB3-mediated regulation of Bergmann glia proliferation in cerebellar lamination. Development. 2015;142(3):522–32. 10.1242/dev.115931 PMC4302995 25564653

