# Supplementary figures and images for: Genetic labeling reveals spatial and cellular expression pattern of neuregulin 1 in mouse brain
Source: Cell Biosci. 2023 May 5;13:79. doi: 10.1186/s13578-023-01032-4 (PMC10161477; doi:10.1186/s13578-023-01032-4)

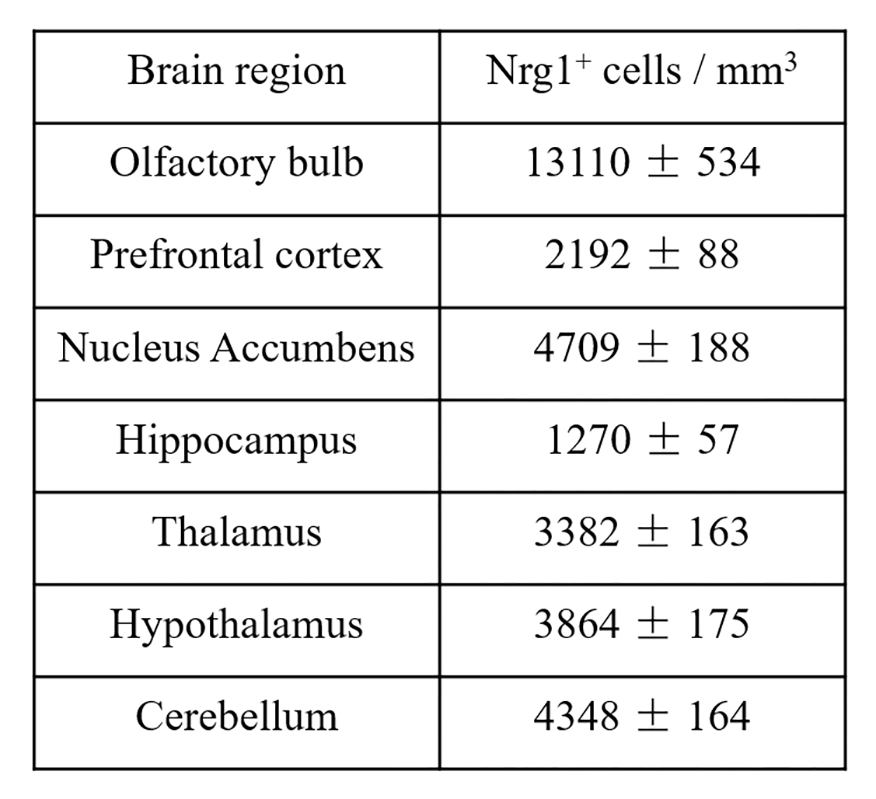

Supplement: Supplementary file 1 — Additional file 1: Figure S1. The densities of Nrg1-positive cells in different brain regions of adult Nrg1-reporting mice. n = 3. [file 13578_2023_1032_MOESM1_ESM.tif]

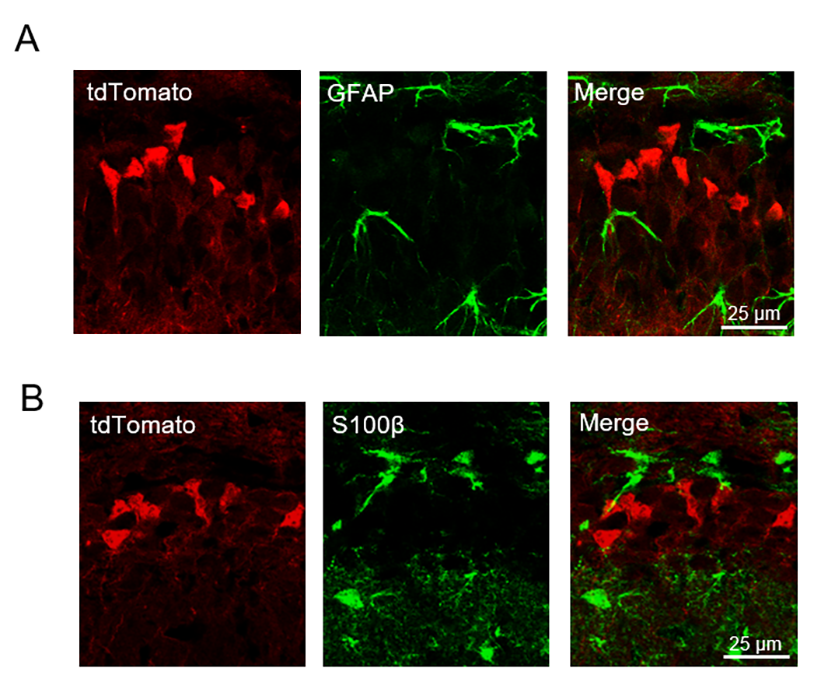

Supplement: Supplementary file 2 — Additional file 2: Figure S2. Nrg1 is not expressed in astrocytes in adult mouse hippocampus. The dentate gyrusof Nrg1-reporting mice were subjected to immunostaining with anti-GFAPor anti-S100βantibodies. Scale bar, 25μm. [file 13578_2023_1032_MOESM2_ESM.tif]
